# Sphingolipids as Regulators of the Phagocytic Response to Fungal Infections

**DOI:** 10.1155/2015/640540

**Published:** 2015-11-25

**Authors:** Arielle M. Bryan, Maurizio Del Poeta, Chiara Luberto

**Affiliations:** ^1^Department of Molecular Genetics and Microbiology, Stony Brook University, Stony Brook, NY 11794, USA; ^2^Department of Physiology and Biophysics, Stony Brook University, Stony Brook, NY 11794, USA

## Abstract

Fungal infections pose a significant risk for the increasing population of individuals who are immunocompromised. Phagocytes play an important role in immune defense against fungal pathogens, but the interactions between host and fungi are still not well understood. Sphingolipids have been shown to play an important role in many cell functions, including the function of phagocytes. In this review, we discuss major findings that relate to the importance of sphingolipids in macrophage and neutrophil function and the role of macrophages and neutrophils in the most common types of fungal infections, as well as studies that have linked these three concepts to show the importance of sphingolipid signaling in immune response to fungal infections.

## 1. Introduction

Beginning in the 20th century, fungi have emerged as important human pathogens. Increases in the population of immunocompromised individuals, due to AIDS or medical interventions, have allowed for invasive fungal infections to take hold in the human population worldwide [1]. Although much work remains to be done in understanding interactions between host and invasive fungi, it is well established that phagocytes serve a central role in the immune response to fungal pathogens [2]. Phagocytes, such as macrophages and neutrophils, are essential effector cells of the innate immune system and are responsible for recognition and killing of fungal pathogens [2–6]. Recent published work has revealed a role for a class of bioactive signaling lipids, known as sphingolipids, in regulating the antimicrobial activity of host phagocytic cells [7–11]. This review will center on the involvement of host sphingolipids in macrophage and neutrophil function during fungal infection. For general reviews on innate antifungal immunity, the reader is referred to [2, 5, 6]. For reviews on microbial sphingolipids in pathogenesis, the reader is referred to [12, 13].

## 2. Invasive Fungal Infections

Unlike bacteria and viruses, systemic fungal diseases were not described until the late 19th century and were considered to be extremely rare. Today, fungal infections are on the rise and there is a pressing need for research focused on immune responses to these relatively “new” human pathogens [14]. It is estimated that there are nearly 1.5 million fungal species; of those species, only a small subset (approximately 300) has been reported to be pathogenic to humans [15]. Although superficial fungal infections, which affect the outer layers of the skin, nails, and hair, are the most common fungal infections in humans, invasive infections pose a more serious threat to human health. Despite the availability of several antifungal drugs, mortality associated with invasive fungal infections remains unacceptably high and is estimated to be over 50% for most mycoses. As a group, fungal infections cause over a million deaths annually worldwide [16]. The most common global opportunistic invasive fungi are* Candida albicans*,* Aspergillus fumigatus*, and* Cryptococcus neoformans*, but there are many other fungal species that infect humans including endemic fungi such as* Blastomyces dermatitidis*,* Coccidioides immitis*, and* Histoplasma capsulatum* [16, 17].

### 2.1. Candidiasis

Candidiasis is caused by commensal* Candida* species, which live in the human gastrointestinal tract and vagina. The most commonly found species is* C. albicans* [5]. In a healthy host, phagocytic cells of the innate immune system are able to recognize and eliminate any invading* Candida* [18]. Under immunosuppressed conditions,* Candida* is able to breach the integrity of mucosal barriers and cause systemic infection. Infection may also occur in patients with a central venous catheter in which* Candida* on the skin is able to bypass cutaneous barriers and a significant amount of fungi enters the circulation [18, 19].* Candida* has the unique ability to switch between yeast and hyphal forms [18, 20]. The ability to reversibly convert from isotropic (yeast) growth to apical (hyphal and pseudohyphal) growth has been theorized to contribute to virulence [21]. Virulence is attenuated in both yeast and hyphal locked mutants and infection sites are populated by both morphological forms, which points to a role for both forms in the pathogenesis of candidiasis [21].

### 2.2. Aspergillosis

*Aspergillus* is ubiquitously found in the environment. The most common pathogenic* Aspergillus* is* A. fumigatus* [22, 23]. Infection occurs via inhalation of conidia into the lungs. Healthy human hosts are typically able to clear invading conidia [21] and prevent germination and spread into the lung [24]. Invasive* Aspergillus* infection occurs primarily when neutrophils are somehow impaired (i.e., chronic granulomatous disease, or neutropenia) and thus unable to contain and clear invasive hyphal growth in the lungs [22–24].

### 2.3. Cryptococcosis

Cryptococcosis is a systemic fungal infection in immune compromised hosts that results in deadly meningitis once the fungus has disseminated to the central nervous system (CNS) [25–27].* C. neoformans*, the most common cause of cryptococcosis, is a yeast commonly found in the environment, and thus exposure is fairly prevalent but rarely progresses to disease in healthy individuals [28–30]. Immunocompetent individuals are able to combat and contain* Cryptococcus* in the lung after inhalation of spores to prevent spread to the CNS. A successful immune response results in killing of* Cryptococcus* by phagocytes and granuloma formation that is thought to prevent* Cryptococcus* from accessing the vasculature and causing infection of the CNS. In the case of an immunocompromised host,* Cryptococcus* is not successfully cleared by phagocytes and spreads through the vasculature and across the blood brain barrier causing life threatening meningitis [26, 30].

### 2.4. Challenges in Development of Therapies against Invasive Fungal Infections

Together, these infections present a unique set of challenges for treatment. Most systemic fungal infections occur in immunocompromised individuals who may be suffering from AIDS, cancer, or organ failure, adding another layer of complexity to the disease [17]. Additionally, there are only a limited number of therapeutic interventions widely available. None of the available classes of drugs are wide spectrum and there is high toxicity associated with the most effective therapies [31]. This relative scarcity of available compounds is owing to the relatedness of fungi to humans compared to viruses and bacteria. Many essential pathways are conserved between fungi and humans, which forces researchers to search for structures and pathways unique to fungi [31]. As an alternative, phagocytes can be exploited as a cell-based therapy in conditions of immune suppression [32, 33]. Therefore, understanding the intracellular pathways that contribute to the killing mechanisms of these immune cells (such as sphingolipid signaling) may provide new means for the development of novel therapeutic strategies against fungal infections.

## 3. Role of Macrophages in Fungal Infections

The name macrophage comes from Greek and means “big eater.” Macrophages are professional phagocytic cells capable of detecting a multitude of signals to bind and consume opsonized pathogens, as well as dying cells and cell debris [34]. Macrophages derive from the myeloid lineage and develop from both monocytic precursors and embryonic progenitors during embryonic development [35]. Tissue macrophages are responsible for immune surveillance and upon recognition of pathogen-associated molecular patterns (PAMPS) will drive inflammation by recruiting other leukocytes including monocytes and neutrophils [36]. They secrete a variety of cytokines including tumor necrosis factor-alpha (TNF-*α*), interleukin 1 (IL1), and nitric oxide (NO), which contribute to activation of antimicrobial defense, and interleukin 12 (IL12) and interleukin 23 (IL23), which direct differentiation of inflammatory T helper cells [37]. They have also been shown to be capable of releasing antimicrobial extracellular traps (ETs) that may play a role in clearance of infections [38].

### 3.1. Candida

Macrophages are one of the most important lines of defense against* C. albicans* in tissues and the blood stream [39]. Evidence for the role of macrophages in* Candida* infection has been demonstrated in different mouse models. For instance, depletion of mouse splenic macrophages (but not neutrophils) with liposome-entrapped clodronate was shown to increase susceptibility of both BALB/cByJ and nude mice strains to disseminated candidiasis [40]. In addition, inactivation of macrophages with intraperitoneal injection of carrageenan was shown to increase susceptibility in an oropharyngeal candidiasis model in BALB/c and CBA/CaH mice [41]. Finally, depletion of alveolar macrophages by 2-chloroadenosine resulted in delayed mortality of BALB/c in a lung injury model but reduced* Candida* clearance and neutrophil recruitment in the lung [42]. From these results, it is thought that macrophages are important for recognition, killing, and recruitment of other cell types but must be activated by T helper 1 cytokines for efficient killing [41]. Macrophages recognize* Candida* through Toll-like receptors 2 and 4, Dectin-1, mannose receptor, and Dectin-2 [18, 43–46]. Mannan has been shown to be one of the most important pattern-associated molecular patterns for recognition of* Candida* by macrophages [18]. Under immune sufficient conditions, the yeast form of* Candida* is effectively controlled by phagocytic action of macrophages, but under certain conditions,* Candida* is able to overgrow and may switch to filamentous hyphal growth that is more of a challenge for the immune system. Once infection is able to take hold (such as under the condition of immune suppression), yeasts that are able to transition to the hyphal form are able to escape macrophages by physically destroying cells due to their size or by inducing pyroptosis [20, 47].

### 3.2. Aspergillus

There is a growing body of evidence for the role of alveolar macrophages in the initial defense against* Aspergillus* conidia that are inhaled into the lung [48]. Alveolar macrophages efficiently uptake* Aspergillus* conidia in a Dectin-1 dependent manner and have the capacity to kill conidia intracellularly [49]. Additionally, invasive aspergillosis has been reported in a patient with inflammatory defective macrophages [50]. Despite this evidence, in 2009, it was reported that macrophages were dispensable in a C57BL/6 mouse model in which clodronate liposomes were used to deplete alveolar macrophages [24]. It was shown that, in the absence of macrophages, neutrophils were capable of mounting a sufficient response to prevent hyphal tissue invasion [24]. Another group has designed transgenic monocyte depleting mice, which utilizes diphtheria toxin induced cell ablation directed toward CCR2 expressing cells. That group reported no difference in lung burden in their depleted mice but showed that they were essential to the priming and expansion of CD4+ T cells [51]. Counter to these results, in 2011, another group has published that depletion of macrophages with clodronate in BALB/c mice results in increased fungal burden in the lung and that the elevated levels of neutrophils failed to control the infection [49]. Altogether, the evidence points to an important supportive role for macrophages in* Aspergillus* infection.

### 3.3. Cryptococcus

In cryptococcal infections, macrophages have been shown to play a critical role in normal host defense but may also have a role in development of disease in immunocompromised individuals. Depletion of macrophages using transgenic diphtheria toxin induced cell ablation directed toward CD11c expressing cells showed increased susceptibility in the mouse model [52]. In an experiment that compared two model hosts, one susceptible (mouse) and one resistant (rat), it was found that clodronate liposome depletion in each species had very different results [53]. While macrophage depletion in mice leads to decreased fungal burden, depletion in rats leads to increased fungal burden and dissemination [53]. Additionally, depletion of alveolar macrophages proved to be protective to immunodeficient mice infected with a glucosylceramide deficient mutant of* Cryptococcus* (Δ*gcs1*) but showed no effect when these same mice were infected with wild type* C. neoformans* H99 [54]. Importantly, the results with the* C. neoformans* Δ*gcs1* strain are of particular clinical relevance since this strain mimics the infection pattern of human cryptococcosis in that it is avirulent in immunosufficient mice and it becomes virulent in T and NK cell deficient mice [54]. Thus, altogether, these findings demonstrate the paradoxical role that macrophages play in cryptococcosis: good cop in case of immunocompetency when macrophages are able to kill the fungus, and bad cop in case of immunosuppression, when they are unable to kill the fungus and rather provide a safe environment for* C. neoformans* to replicate and be transported elsewhere (favoring dissemination). Indeed, in immunocompetent subjects, clearance of internalized* Cryptococcus* is thought to depend on T helper 1 mediated response which results in formation of a granuloma and production of TNF-*α* and Interferon gamma (IFN*γ*) [55]. These cytokines cause macrophages to become classically activated and upregulate NADPH oxidase to allow for production of nitric oxide which kills internalized* Cryptococcus* [56]. On the other hand, in an immunocompromised host,* Cryptococcus* is able to survive and proliferate within macrophages leading to eventual dissemination into the blood stream and central nervous system [26, 57]. There is further evidence for this transcellular passage theory, also known as “Trojan horse” model. An experiment which inoculated mice with macrophages already containing* Cryptococcus* showed increased fungal burden in the lung and spleen and also the brain at later stages of infection as compared to mice inoculated with the same number of free yeasts [58]. It was also shown that late stage depletion of macrophages (72 hours after intravenous infection) resulted in decreased disease severity and fungal burden [58]. As another way to subvert macrophage processes and disseminate,* Cryptococcus* has also been shown to extrude itself from macrophages, leaving both macrophage and yeast intact [59]. Altogether, this evidence supports a protective role for macrophages in an immunocompetent host but strongly supports the subversion of macrophages in the condition of immunosuppression resulting in increased dissemination. Generally, an efficient uptake of* Cryptococcus* by macrophages requires the opsonization by complement or specific antibodies [60, 61] while the presence of a large capsule on* Cryptococcus* prevents phagocytosis* in vitro*.

## 4. Role of Neutrophils in Fungal Infections

Neutrophils are considered to be the most important cell type for fungal killing. They sense pathogens with an array of pattern recognition receptors (PRRs), which include Toll-like receptors, C-type lectin receptors, glycosphingolipids (GSLs), and cytoplasmic sensors for ribonucleic acids [62, 63]. PRRs, along with signals from other immune cells (such as macrophages), work together to help neutrophils sense their environment, undergo chemotaxis, and initiate inflammatory responses [62, 64, 65]. Neutrophils are equipped with an arsenal of granule proteins that have various enzymatic activities designed to neutralize pathogens, including defensins, myeloperoxidase, proteases, lactoferrin, and gelatinase [65, 66]. Once activated, neutrophils carry out effector functions, which include phagocytosis, mobilization of granules, production of reactive oxygen species (ROS), release of neutrophil extracellular traps (NETs), and secretion of lytic enzymes, antimicrobial peptides, and neutrophil derived cytokines. These activities ultimately lead to pathogen destruction by both intracellular and extracellular killing and recruitment of additional immune cells [64–66].

### 4.1. Candida

Neutrophils are thought to be critical for controlling systemic candidiasis. Patients suffering from induced neutropenia or genetic neutrophil defects are at high risk for invasive* Candida* infection [67, 68]. In the mouse, ablation of neutrophils using RB6-8C5 (anti-Gr-1, anti-Ly6G/Ly6C) antibody causes increased susceptibility to systemic, vaginal [69], and oropharyngeal challenge with* Candida* [41]. Three mechanisms have been described by which neutrophils kill* Candida* in healthy individuals. The first is killing of unopsonized* Candida* and it depends on complement receptor 3 (CR3) and caspase recruitment domain-containing protein 9 (CARD9). A second mechanism of killing targets opsonized* Candida* in an Fc*γ* receptor (Fc*γ*R), protein kinase c (PKC), and NADPH oxidase dependent manner [67]. Finally, a third mechanism involves a newly discovered function of neutrophils in the generation of neutrophil extracellular traps (NETs). NETs are weblike structures extruded by neutrophils composed of decondensed chromatin and over 30 different neutrophil proteins [70]. NETs are generated in response to* Candida* hyphae [71] and contain the antifungal protein calprotectin [72]. It is thought that while intact neutrophils are able to clear yeast forms of* Candida*, NETs may have evolved as a way to defend against hyphae that evade phagocytosis due to their size [71].

### 4.2. Aspergillus

Neutrophils are essential to defend the host against* Aspergillus* infection. Like* Candida* infection, neutropenia and neutrophil defects (such as chronic granulomatous disease) are major risk factors for invasive aspergillosis [73]. It has been confirmed that depletion of neutrophils via monoclonal antibody RB6-8C5 (anti-Gr-1, anti-Ly6G/Ly6C) during the earliest phase of infection is associated with high mortality which shows that neutrophils provide essential defense during inhalation and germination of* Aspergillus* [24]. It is still unclear how neutrophils control* Aspergillus* in healthy individuals. One theory is that neutrophils spread and degranulate onto the surface of hyphae [74]. New research suggests that NETs may also play a role. NETs are formed in response to* Aspergillus* hyphae [71] and restoration of NET formation using gene therapy to add the gp91(phox) gene (encoding a subunit of NADPH oxidase) in a patient with chronic granulomatous disease was shown to rapidly cure aspergillosis [73].

### 4.3. Cryptococcus

Although macrophages are considered the first line of defense against* C. neoformans*, the role of neutrophils is equally important because, once recruited, they are extremely efficient in killing* C. neoformans* and other fungal cells [75, 76]. Studies on the role of neutrophils during* C. neoformans* infection have not been pursued much, mainly because primary neutropenia is not a risk factor for cryptococcosis. However, this does not mean that neutrophils are not important for protection against cryptococcosis, and it only suggests that the decrease of neutrophils is not sufficient to render the host susceptible to* C. neoformans*. On the other hand, neutrophils might play an important role for protection once the infection has occurred. This is exemplified by many observations. First, patients in which neutrophil killing activity is decreased may actually develop cryptococcosis [77, 78]. Second, in late stages of human immune deficiency virus (HIV) infection, with low number of CD4+ T cells and when cryptococcosis occurs, the defensive mechanisms of macrophages and neutrophils are depressed [79]. Thus, it is largely accepted that most, if not all, opportunistic infections in acquired immune deficiency syndrome (AIDS) patients (including cryptococcosis) also develop because neutrophils and macrophages are not fully activated [79, 80]. Third, macrophage-mediated chemotaxis, phagocytosis, production of cytokines, superoxide, extracellular traps, and antimicrobial peptides and their killing activity are not optimal in the late stages of AIDS [80–83]. Fourth, although it is reported that cryptococcosis is not usually associated with human neutropenia or defective neutrophil function, neutropenia is often present in HIV positive patients, especially when patients have been diagnosed with AIDS [80, 81, 84]. Fifth, there are also reports showing that apparent immunocompetent individuals with pulmonary cryptococcosis have impaired killing activity of neutrophils and monocytes due to deficient production of TNF-*α*, IL-1*β*, and nitric oxide [77]. These studies clearly highlight that neutrophils are important to control* Cryptococcus* infection in humans.

**Figure 1:**
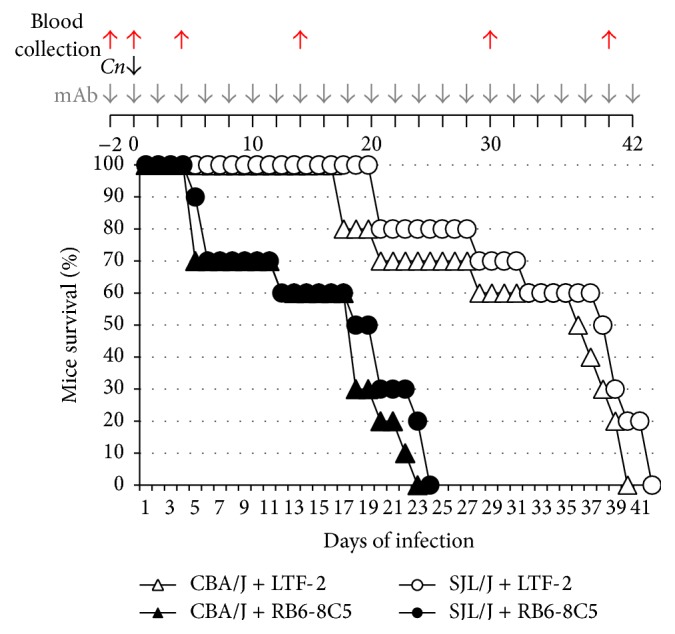
Neutrophils are important to control cryptococcosis in mice. Four six-week-old mice (CBA/J or SJL/J model) were treated intraperitoneally every other day with 300 *μ*g of Rb6-8C5 monoclonal antibody (mAb, gray arrows) directed against neutrophils. After 2 days from the first dose (day 0), mice were infected intranasally with a lethal dose of* C. neoformans* cells (5 × 10^5^) (black arrow). As controls, mice were treated with LTF-2 mAB (an IgG2 isotype for Rb6-8C5). Before mAb treatment and* C. neoformans* challenge, and during infection, blood was collected for neutrophil count (red arrows).

Studies in mice are controversial mainly because murine neutrophils are notoriously weak compared to humans as they do not produce (and secrete) fully activated defensins [85]. Consequently, the role of neutrophils in* C. neoformans* infection is still unresolved: only a very limited amount of published work has addressed this issue using animal models and depending on the model used (mouse and/or* C. neoformans* strains and/or size and route of the inoculum) the results seem to differ [52, 86–88]. For instance, Casadevall's group found that depletion of neutrophils in BALB/c mice infected with the weak* C. neoformans* strain D52 (a mouse model in which mice succumb to the infection) enhanced resistance of the host [86] whereas other* in vivo* studies have implied a protective role for neutrophils when a mouse strain (SJL/J) relatively resistant to cryptococcosis was employed [88]. In the first model of infection (BALB/c mice with D52* C. neoformans*), depletion of neutrophils before intratracheal* Cryptococcus* instillation resulted in protection of mice [86]. In this study, however, only a single depletion of neutrophils (effective for approximately 3 days) was performed; indeed, at day 7 of infection, neutrophil numbers were up again to the level of control mice. This early and short window of intervention points to a damaging role of neutrophils during the initial phase of the infection [52, 86] and it does not allow for formulating an overall conclusion regarding the role of neutrophils in the final outcome of* Cryptococcus* infection. Considering that neutrophils continue to accumulate considerably also in the later phases of infection [86, 88], the question remains as to whether neutrophils exert different roles in different stages of the disease especially before an effective T cell mediated response is mounted (2-3 weeks). To definitively assess the role of neutrophils during cryptococcosis, we depleted neutrophils throughout the infection, in two different mouse strains (CBA/J or SJL/J) infected with a clinical isolate and highly virulent* C. neoformans* (H99) (Figure 1). Neutrophils were depleted by injecting 300 *μ*g of RB6-8C5 monoclonal antibody intraperitoneally, as indicated (Figure 1). Confirmation of neutropenia, defined here as a decrease of at least 70% of neutrophils, was confirmed before* Cryptococcus* challenge and throughout the survival experiment by blood neutrophil count. In our hand, 300 *μ*g of RB6-8C5 was the minimum dose required to ensure the 70% decrease of neutrophils. As a negative control, 300 *μ*g of LTF-2 isogenic mAb was administered using a similar dose regime and neutrophils were also routinely counted in these mice and no depletion was found. Mice were then challenged with* C. neoformans* H99 strain intranasally and survival was monitored and recorded. The average survival of CBA/J and SJL/J neutropenic mice was 15.4 ± 7 and 16.8 ± 7.8, respectively, whereas the average survival of nonneutropenic mice was 32.2 ± 9.7 and 35 ± 8.5, respectively (*P* < 0.05) (Figure 1). These results clearly indicate that neutrophils are important to control* Cryptococcus* infection in mice. In line with our novel observations, other mouse models also supported a protective role for neutrophils [88, 89]. In one model, which employed the rather resistant mouse strain SJL/J (similar to CBA/J) infected with the* C. neoformans* strain D52, the T helper 1 response was preceded by accumulation of neutrophils in the lung as early as 3 hours after infection together with increased macrophage inflammatory protein-1*α* (MIP-1*α*), monocyte chemotactic protein 1 (MCP 1/CCL2), and keratinocyte chemoattractant (KC), which are neutrophil and macrophage chemoattractants. The number of neutrophils in the lung progressively and greatly increased in the following days and weeks while the fungal burden decreased [88]. In another study,* in vivo* imaging was used to show neutrophils directly removing* C. neoformans* from the brain vasculature [89]. Additionally, it was shown that depletion of neutrophils enhanced fungal burden in the brain [89]. Thus, from these studies, it is obvious that the apparent conflicting results in the literature are likely due to the use of different mouse models,* Cryptococcus* strains, and most importantly the time frame of the induced neutropenia. Altogether, these studies and our new results (Figure 1) strongly point to the fact that neutrophils are important to control* Cryptococcus* infection, especially when the infection has already developed.

## 5. The Role of Sphingolipids in the Immune Responses

Sphingolipids are a family of lipids defined by a backbone mostly composed of an eighteen-carbon amino alcohol, referred to as the sphingoid backbone. The simplest sphingolipids are sphingosine, phytosphingosine, and dihydrosphingosine, which can be modified to produce an array of more complex sphingolipids, some of which have regulatory functions in important cell processes. For general reviews on sphingolipid metabolism and signaling, the reader is referred to [90–94].

**Figure 2:**
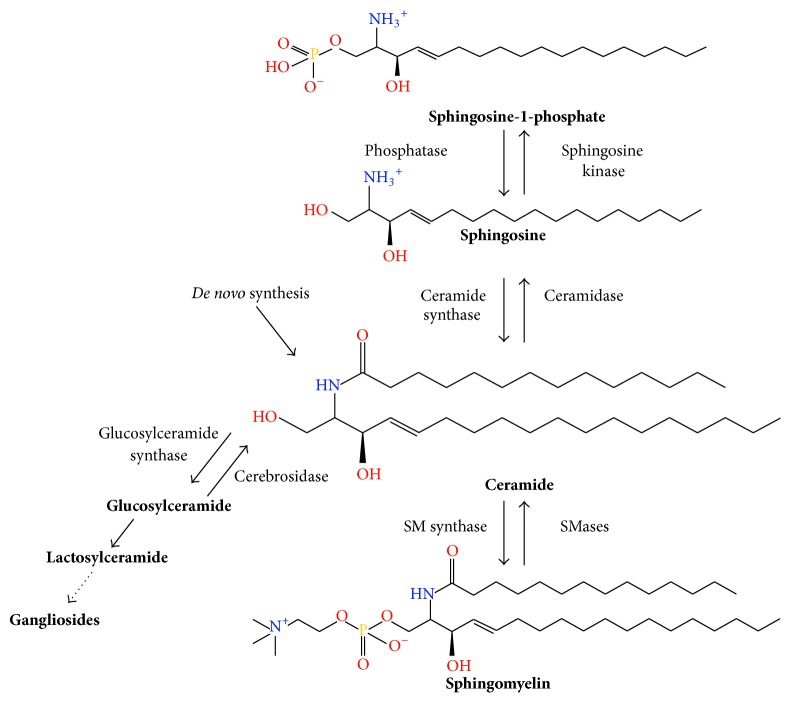
Overview of sphingolipid synthesis. Sphingolipids contain a sphingosine backbone that is modified to produce an array of metabolites. Ceramide serves a central role and can be synthesized by breakdown of sphingomyelin, addition of fatty acid by ceramide synthase, or* de novo* synthesis from serine palmitoyltransferase. Ceramide and sphingosine can be phosphorylated by their respective kinases to form bioactive metabolites. More complex sphingolipids are formed from ceramide, including sphingomyelin and glycosphingolipids.

Among the bioactive sphingolipids that have been implicated in the regulation of the immune response against fungal infections are sphingosine-1-phosphate (S1P), sphingomyelin (SM), and glycosphingolipids (GSLs) (Figure 2) [94].

### 5.1. Sphingosine-1-Phosphate

S1P is produced by the phosphorylation of sphingosine by one of two sphingosine kinases (SK1 and SK2) [94]. Once phosphorylated, S1P is recognized by a family of G-protein coupled receptors (S1PR1-5) that activate downstream effectors such as small GTPases (Rho, Rac, and Ras), adenylate cyclases, PI-3-kinase, phospholipase C, protein kinase C, or intracellular calcium [91]. The distribution of the receptors on different cell types and the coupling of receptors to different G-proteins allow S1P to differentially exert its influence in many different pathways, including inflammation [95]. S1P may also signal independently of S1PRs as an intracellular second messenger [96].

### 5.2. Sphingomyelin

SM is produced by the addition of a phosphocholine moiety from phosphatidylcholine (PC) onto ceramide by a family of enzymes known as sphingomyelin synthases. In mammals, there are two sphingomyelin synthases, SMS1 and SMS2. SM is an abundant component of cell membranes and is important for the formation of ordered membrane domains known as lipid rafts in model membranes [97, 98]. It is thought that lipid rafts play important roles in many processes such as GPI-anchored protein sorting, receptor clustering [99], endocytosis, exocytosis, vesicle formation, and budding [100, 101]. Thus, the ability of SM to contribute to lipid raft homeostasis may have important implication in the functions of phagocytes whose activities rely on receptor activation, endocytosis, and secretion. So far, it has been shown that SMS2 deficiency prevents TNF-*α* stimulated lipid raft recruitment of TNF receptor 1 and prevents NF*κ*B activation in macrophages [102]. Additionally, SM can also be broken down by the sphingomyelinase (SMase) enzymes to produce ceramide and phosphocholine, thus serving as a major source of the bioactive sphingolipid, ceramide [103, 104]. During synthesis of SM, SMSs also produce the bioactive product diacylglycerol (DAG) [105–107] which can activate DAG-binding targets, such as protein kinase D (PKD). Indeed, PKD is a key regulator of protein trafficking and secretion, and it has been shown to control neutrophil secretion of antifungal factors [8, 105].

### 5.3. Glycosphingolipids

GSLs are composed of a sugar moiety attached to ceramide. More than 400 types of GSLs have been identified based on the attached sugar structure, but the ceramide chain lengths are also highly variable [108, 109]. Glycosphingolipid biosynthesis occurs via the action of specific glycosyl transferases, which add galactose or glucose moieties to ceramide [94]. These can be further modified to produce an array of carbohydrate structures [110]. Major relevant GSL species in phagocytes include lactosylceramide and gangliosides [10, 111, 112]. GSLs are another major component of lipid rafts and have also been found to have direct interaction with both cytosolic and membrane proteins; they play roles in cell adhesion, motility, growth, and neutrophil function [111, 113–115]. Importantly, GSLs have been shown to be able to directly bind to pathogens which is a crucial step in initiating phagocytosis [111, 116, 117]. For example,* Chlamydia pneumoniae* and* Chlamydia trachomatis* have been shown to bind both Asialo-GM2 and GM1 [118], while influenza virus binds poly_(→50)_ glucosylceramides and other GSLs [119]. For a thorough discussion on the topic, please refer to [116].

## 6. The Role of Host Sphingolipids in Fungal Infections

### 6.1. Candida

There is evidence for the role of host sphingolipids in the regulation of the immune response to* Candida*. It has been shown that inhibition of sphingosine synthesis with myriocin in* Galleria mellonella*, a commonly used insect model for studying fungal infections [120], increases mortality during* Candida* infection [121]. In the mouse model, sphingolipid synthesis inhibition with myriocin or fumonisin B1 treatment impairs phagocytosis of* C. albicans* by macrophages in culture [122]. Fumonisin B1 treatment of mice increased susceptibility to tail vein injected* C. albicans* [122]. Additionally, the importance of the GSL lactosylceramide (LacCer) in neutrophil function has been studied and it was reported that LacCer is expressed on the plasma membrane of neutrophils [10, 115]. It is important for superoxide generation and the formation of domains with the Src family kinase Lyn [114, 115]. These observations are important in light of the evidence supporting the role of neutrophils in* Candida* infection. Furthermore, LacCer can bind* Candida* directly [123] and it also acts as a pattern recognition receptor to promote chemotaxis of neutrophils in response to* Candida* soluble beta-D-glucan [63]. Additionally, GSLs and specifically gangliosides have been shown to play essential roles in adhesion and motility, both important processes for phagocytes to serve their function [113].

More recently, sphingolipids have been implicated in the production of NETs. Neumann et al. demonstrated that treatment of primary blood-derived human neutrophils with bacterial sphingomyelinase, which hydrolyzes SM into ceramide and phosphocholine, causes spontaneous generation of NETs [124]. Although the mechanism for this observation is unknown, the breakdown of SM could alter signaling complexes that localize to rafts and lead to spontaneous NET generation. This observation points to a role for rafts in controlling the generation of NETs and suggests that SM and GSL pathways could contribute to clearance of* Candida* by NETs. Since the importance of neutrophils and macrophages for fighting* Candida* infections is well established, these insights into sphingolipid involvement in phagocyte function could aid in developing alternative therapeutic strategies against this fungus.

### 6.2. Cryptococcus

Host sphingolipids have been shown to play an important role in controlling* Cryptococcus* infections. In particular, S1P plays a role on multiple levels. In an obligate intracellular murine model of* Cryptococcus* infection (Δ*gcs1*), which forms granulomas, SK1, the enzyme responsible for production of S1P, was found to be essential to granuloma formation. In fact, knockout of SK1 prevented formation of granulomas by reducing the amount of S1P in the bronchoalveolar lavage fluid which resulted in lowered levels of MCP-1 and TNF-*α* [9, 25]. Additionally, S1P was found to directly affect phagocytic cells. While addition of S1P to macrophages increased their ability to uptake* Cryptococcus* via the action of S1P receptor 2 [125], addition of S1P to neutrophils increased their ability to kill* Cryptococcus* extracellularly [9]. Sphingomyelin may also play a role in regulating the response of phagocytic cells to* C. neoformans*. In fact, some work has hinted at a role for lipid rafts in phagocytosis of* Cryptococcus* as disruption of lipid rafts with methyl-*β*-cyclodextrin results in decreased uptake of* Cryptococcus* by macrophages* in vitro* [126]. Since SM and glycosphingolipids are key constituents of lipid rafts, these studies warrant further investigation on the requirements also for these complex sphingolipids in the recognition and phagocytosis of* C. neoformans* by macrophages [97]. Finally, inhibition of SMS, the enzyme responsible for SM biosynthesis, impairs the killing ability of neutrophils by preventing the release of antifungal factors through a DAG-PKD dependent mechanism [8, 105].

### 6.3. Aspergillus

There is a dearth of information concerning host sphingolipid involvement in* Aspergillus* infection. It is known that neutrophils and NETs play an important role in clearance of infection. As discussed in the previous sections, sphingolipids are important for many neutrophil antifungal activities, including secretion of antifungal factors, and possibly regulating NET formation. This warrants further study to extend work that has been done in other fungi to include* Aspergillus* and other emerging fungi.

### 6.4. Other Fungal Infections

There is an increasing amount of evidence that lipid rafts play a role in the interaction between phagocytes and fungi. Both complement receptor 3 and Dectin-1 are major fungal pattern recognition receptors and they have been shown to colocalize in lipid raft microdomains in response to* Histoplasma capsulatum* [127]. This finding shows the importance of these sphingolipid rich domains especially during fungal infections, many of which are recognized through these receptors.

## 7. Conclusions and Future Directions

Sphingolipids have been shown to play an important role in many cellular processes, including the function of phagocytic cells, which play critical roles in invasive fungal infections. Signaling lipids such as S1P are able to directly bind proteins to affect cellular pathways, while SM and GSLs may affect cellular processes by altering domain formation on the plasma membrane or serving as pattern recognition receptors themselves (LacCer). Findings that highlight the roles of sphingolipids in phagocytes are particularly useful in light of the critical role that these cells play in controlling fungal infections and may serve as a key to overcome the challenges associated with treating these types of infections. In the future, it is important to apply what we learned about phagocytes into understanding how sphingolipids affect the interactions between phagocytes and fungi. Much work that has been done concerning this has not yet been validated for other species. Another unexplored pathway is the possible connection between sphingolipids and formation of extracellular traps and whether this could be another avenue to fight off hyphal growth. In the future, understanding of host pathways in phagocytes could lead to cell-based therapies that exploit the strengths of phagocytes to combat fungal infections in the context of an immunocompromised system.
